# Foreign body in ear, nose and oropharynx: experience from a tertiary hospital

**DOI:** 10.1016/S1808-8694(15)30052-5

**Published:** 2015-10-19

**Authors:** Romualdo Suzano Louzeiro Tiago, Daniel Cauduro Salgado, Juliano Piotto Corrêa, Márcio Ricardo Barros Pio, Ernani Edney Lambert

**Affiliations:** a MD, MS, PhD in Otorhinolaryngology - UNIFESP. Assistant Physician at the Hospital do Servidor Público Municipal de São Paulo.; b MD, 2nd year resident of otorhinolaryngology - Hospital do Servidor Público Municipal de São Paulo.; c MD, 2nd year resident of Otorhinolaryngology - Hospital do Servidor Público Municipal de São Paulo.; d MD, 3rd year resident of otorhinolaryngology - Hospital do Servidor Público Municipal de São Paulo.; e MD, 3rd year resident of otorhinolaryngology -Hospital do Servidor Público Municipal de São Paulo.

**Keywords:** Ear, Nose, Oropharynx

## Abstract

The ocurrence of foreign bodies in otorhinolaryngology is reason of constant searches for emergency services. **Objective:** To value the incidence of patients with foreign body, to analyze the clinical situation and the treatment in these cases. **Method:** The prospective study was realized in 81 patients with diagnosis of foreign body of nose, ear or oropharynx in the otorhinolaryngology service of the Hospital do Servidor Público Municipal de São Paulo between april/2003 and march/2005. **Results:** 57 cases of foreign body of ear, 13 cases of nose and 11 of oropharynx. These patients, 51.85% were men and 48.15% were women. The age average was 23 years old. The average of the evolution time was 18.36 days, being that 38.27% these cases were taken care in less 24 hours of evolution. Inside the total of patients, 83.95% received initial attendance in the otorhinolaryngology clinic, and 16.05% came of another service after some previous removal attempt. The most common symptom of the foreign bodies cases of oropharynx it was odinofagia present in 90.91% of the cases; in the foreign bodies of nose, the unilateral rhinorrhea and cacosmia were present in 46.15 of the cases; in the foreign bodies of ear, 38.60% evolved without symptoms and 28.07 with hipoacusia. The most frequent foreign body of oropharynx it was the fish spine (54.55%); in the nose it was the paper (30.77%); and in the ear it was the cotton (31.58%). The complications resulting of the presence of foreign body or about the manipulation of these had been found in 13 cases (16.05%). **Conclusion:** Most cases of foreign body conditions, in which a non-specialist professional or a non-professional person previously handles its removal, have a bad evolution with emerging complications. Such outcomes strengthen the fact that an otorhinolaryngologist using the proper equipment must treat patients with foreign body.

## INTRODUCTION

Foreign bodies (FBs) in the nose, ears and the oropharynx are reason for frequent visits to otorhinolaryngology emergency units. FB cases rarely go without symptoms, which are determined by the time or duration the FB stays in place before removal. In the nose initial symptoms are sneezing, serous coryza and nasal obstruction, eventually progressing after a few days to unilateral foul smelling purulent rhinorrhea. In the ear initial symptoms may be hypoacusis, otorrhagia, otorrhea or buzzing and the diagnosis may be confirmed by otoscopy. The main symptom in oropharynx FBs is odynophagia^1^.

FBs may vary widely in shape, size and composition. Schulze et al. (2002), in a retrospective study of 698 ear FB cases, proposed an 8-class classification system based on FB shape and texture^2^. FBs may also be classified according to the introduction mode, either voluntary or accidental. Voluntary placement of FBs occurs mostly in children and accidental introduction is usually of live animals^3^.

The shape and size of FBs establishes the level of difficulty in their removal. In ear FBs complications may arise as the external auditory canal is small and important anatomical structures are close.4 Removal may be difficult when the FB is close to the tympanic membrane or the bony external auditory canal because of intense pain due to increased sensitivity. Frequent complications include: laceration of the external auditory canal, tympanic perforation, external otitis and hematomas. FBs in the nose may progress with epistaxis, septal perforation and rhinosinusitis depending on the time the FB has been in place and its location^1^. Complications may also arise frequently during FB removal, making it essential for ENT specialists to treat these cases.

Bressler et al. (1993) reviewed prospectively 98 ear FB cases, concluding that FBs not removed by primary medical care should be referred to expert evaluation^4^. Ikino et al. (1998) reviewed 88 ear and nose FB cases in pediatric patients and suggested that prevention is the preferred option and that parents and relatives should be informed about the risks of foreign objects introduced in the ear and nose.6 Marques et al. (1998) conducted a prospective review of 477 nose, ear and oral cavity FB cases and noted a high complication rate in those cases where removal was attempted by untrained or unprepared professionals^1^. In a retrospective review of 162 ear FB cases, Thompson et al. (2003) concluded that successful FB removal depends on patient cooperation, the ability of the physician to see the FB, the type of FB, prior manipulation and available instruments^5^.

The aim of this study was to establish the incidence of patients with FBs in the otorhinolaryngology unit at the Sao Paulo Municipal Servidor Publico Hospital (HSPM) (Hospital do Servidor Publico Municipal de Sao Paulo) and to study the clinical findings and the treatment of these patients.

## MATERIAL AND METHODS

A prospective study of 81 patients admitted to the HSPM Otorhinolaryngology Unit between April 2003 and March 2005 with a diagnosis of foreign objects in the nose, ear or oropharynx that had underwent a complete otorhinolaryngological examination followed by FB removal. The initial clinical assessment and the FB removal procedure were performed by one of the resident physicians monitored by a senior physician of the HSPM Otorhinolaryngology Unit.

A specific protocol form was filled in for all cases, including the following points: age; main complaint; associated symptoms; duration of the condition; location; nature of the FB; strategy and complications. Data were collected by the ENT specialist who was responsible for the patient, based on the interview of the patient or child caretaker.

One of the following instruments was used to remove ear FBs: a curette, an alligator forceps, ear syringing or irrigation and bayonet forceps. In selected cases a microscope was used to facilitate FB handling. Instruments used for nasal FB were: Itard’s catheter, alligator forceps or a curette. Instruments used to remove pharyngeal FBs were: a Hartmann forceps a bayonet forceps or digital maneuvers, always with local anesthesia with 10% lidocaine. Alcohol was instilled in the external auditory canal prior to removing live FBs. General anesthesia was required in only one patient to remove the FB.

## RESULTS

Our study included 81 cases of otorhinolaryngological FBs: 57 (70.37%) in the ear, 13 (16.05%) in the nose and 11 (13.58%) in the oropharynx. Age varied from 2 to 84 years (average 23 years). The 0-3 year group was the most frequently affected groups for nose FBs, with 11 cases (84.61%). The 4-20 years group was the most frequently affected group for ear FBs, with 27 cases (47.37%). Pharyngeal FBs were more frequent in patients over 20 years of age, with 9 cases (81.82%) (Table 1).

**Table 1:** Frequency of cases with foreign bodies according to the age group and the site of the foreign body.

AGE				
SITE	0-3 years	4-20 years	>20 years	Total
Ear	6	27	24	57
Oropharynx	1	1	9	11
Nose	11	2	0	13
Total	18	30	33	81

Gender distribution was 42 men (51.85%) and 39 women (48.15%) (Table 2). The average FB duration time was 18.36 days; 31 cases (38.27%) were seen within 24 hours of FB duration. Foreign objects in the ear cases had an average FB duration time of 14.24 days. Foreign objects in the nose cases had an average FB duration time of 49.23 days. Foreign objects in the oropharynx cases had an average FB duration time of 1.72 days. First treatment was given in the otorhinolaryngology clinic in 83.95% of patients; 16.05% were referred from other clinical units following some attempt at removal.

**Table 2:** Frequency of cases with foreign bodies according to gender and the site of the foreign body.

GENDER		
SITE	Male	Female
Ear	32	25
Oropharynx	5	6
Nose	5	8
Total	42	39

The location of FBs was as follows: of 57 ear FBs, 29 (50.88%) were placed in the right ear, 24 (42.11%) in the left ear and 4 (7.02%) in both ears. Nasal FBs were 8 cases (61.54%) in the right naris and 5 (38.46%) in the left naris. Of 11 pharyngeal FB cases 4 (36.36%) were in the right tonsil, 4 (36.36%) in the left tonsil and 3 (27.27%) at the base of the tongue.

The most common symptom in oropharyngeal FBs was odinofagia in ten cases (90.91%). Unilateral rhinorrhea and cacosmia were found in six cases (46.15%) of nasal FBs, followed by unilateral rhinorrhea in four cases (30.77%). No symptoms were found in 22 cases (38.60%) of ear FBs; 16 (28.07%) developed hypoacusis and 9 (15.79%) presented otalgia (Table 3).

Of 57 patients with ear FBs, 23 (40.35%) were removed with alligator forceps, 18 (31.58%) with irrigation, 8 (14.04%) with a curette and 8 (14.04%) using more than one method. Of 13 patients with nasal FBs, extraction was done using Itard’s catheter in 9 (69.23%), alligator forceps in 2 (15.38%) and more than one method in 2 (15.38%). Of 11 pharyngeal FBs, 7 (63.64%) were removed with a Hartmann forceps, 2 (18.18%) with a bayonet forceps and 2 (18.18%) using digital maneuvers.

FBs were classified as animate organic, inanimate organic and inorganic. There were 49 (85.96%) inanimate organic FBs in the ear, 5 (877%) inorganic FBs and 3 (5.26%) animate organic FBs. There were 12 (92.31%) inanimate organic FBs in the nose and 1 (7.69%) inorganic FB. There were 11 (100%) inanimate organic FBs in the pharynx.

The most frequent FB in the oropharynx was fish spine, with 6 cases (54.55%). Paper was the most frequent object in the nose, with 4 cases (30.77%), followed by sponges, with 3 cases (23.08%). The most frequent FB in the ear was cotton, with 18 cases (31.58%), followed by plastic, with 7 cases (12.28%) (Table 4).

Complications resulting from FBs and previous removal attempts were seen in 13 cases (16.05%). Oropharyngeal FBs did not present complications. Seven ear FB cases (12.28%) presented with acute external otitis and one case (1.75%) had a laceration in the external auditory canal. Three nasal FB cases (23.08%) presented with rhinosinusitis; one (7.69%) had epistaxis and one (7.69%) had a rhinolith.

## DISCUSSION

In this study there was a slight predominance of FBs in men (51.85%) compared to women (48.15%), similar to results published by other authors.5 The average age was 23 years, similar to Bressler’s et al. review (1993)^4^. Nose FBs were more frequent in children, particularly in the 0-3 years age group (84.61%), as seen in another review^1^. The incidence fell with age; no FBs in the nose were seen in patients over 20 years of age. This reflects the sense of curiosity and self-discovery typical of children. With growth and cognitive development, placing FBs in the nose becomes rare and is seen only in psychiatric patients^6^.

**Chart 1:**
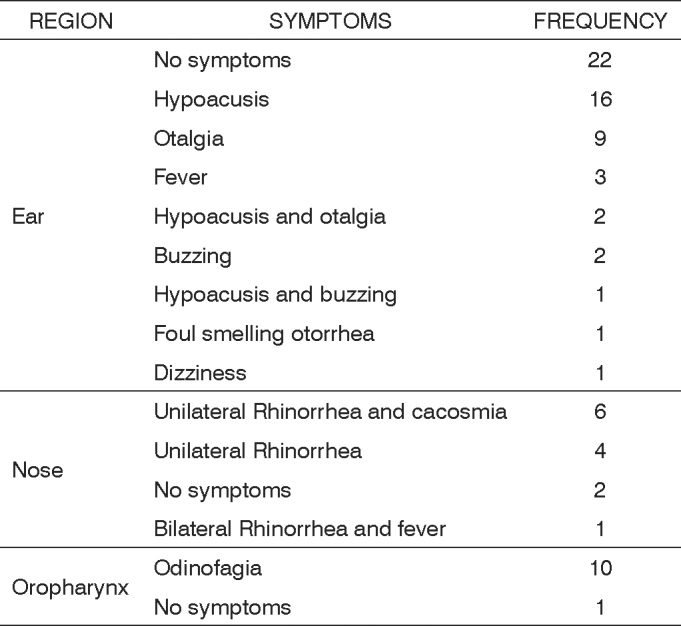
Symptoms related to foreign objects according to the site.

**Chart 2:**
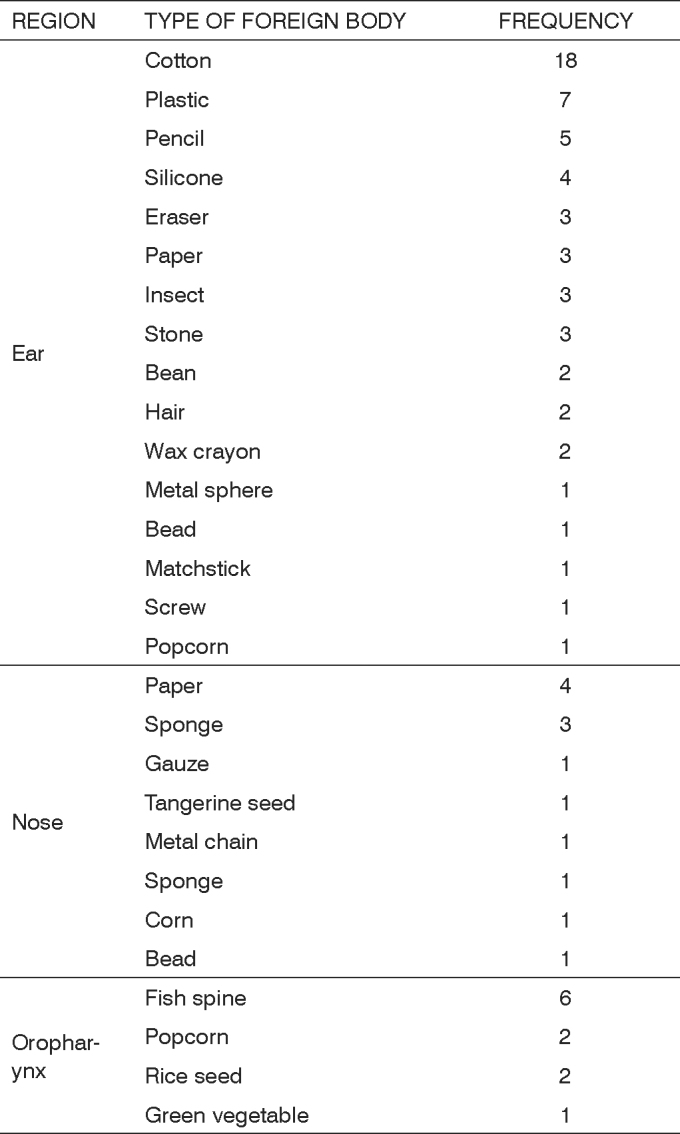
Frequency of the FB type according to the site.

On the other hand, oropharyngeal FBs were more frequent in patients over 20 years of age (81.82%). Only two pediatric patients in this category were seen at our Otorhinolaryngology Unit, probably due to the fact that these patients usually are referred directly to the Endoscopy Unit due to the risk of airway obstruction^6^.

Ear FB cases had a balanced distribution between the 4-20 years age group (27 cases) and the over 20 years age group (24 cases). FB placement in the 4-20 years age group was seen mostly between 3 and 12 years of age (23 cases), which may be explained by play between children. FB were introduced accidentally in patients over 20 years such as during the act of scratching the ear with cotton (15 cases) or by introducing ear plugs (4 cases). The three cases in which the FB was an insect in the external acoustic canal were over 12 years of age.

FB duration in the specific site was less than 24 hours in 38.27% of cases, similar to the study by Ikino et al. (1998)^6^. The average FB duration time in the oropharynx was only 1.72 days, explained by the resulting discomfort due to the FB in this site. FBs in the nose, however, had an average FB duration of 49.23 days due to a rhinolith in a 10-year-old child in which a sponge fragment remained approximately 510 days in the nasal cavity. Rhinolithiasis is a rare disease, characterized by coral-like calcium concretions deposited progressively around a FB,7 uncommon in pediatric patients^8^. The average ear FB duration time was 14.24 days. In symptomatic ear FB cases, the average FB duration was 20.08 days. Average FB duration in asymptomatic ear FB cases (38.60%) was 4.86 days.

16.05% of the total number of patients was referred to our unit following an unsuccessful attempt at FB removal at another unit. This percentage is lower compared to other studies. This may be explained by the fact that municipal civil servants first seek medical services at the HSPM (a city hospital for civil servants). There is also a good relationship between the Emergency Room and the Pediatrics Department staff and our Otorhinolaryngology Unit.

Oropharyngeal FBs were found in the tonsils or at the base of tongue, probably due to tonsillary crypts that favor food retention. Nose and ear FBs are more frequent to the right. We believe this is due to the fact that most of the population is right-handed. Thompson et al. (2003) noted only a minor predominance of FBs in the right ear (52%)^5^.

The most frequent symptom in oropharyngeal FB cases was odinofagia (90.91%) which motivated patients to seek medical help earlier (1.72 days). The most frequent symptom in nose FB cases was unilateral rhinorrhea, in 76.92% of patients. The absence of symptoms was the most common presentation in ear FB cases (38.60%) and the most frequent symptom was hypoacusis (28.07%).

Removal methods most commonly used for ear, nose and oropharyngeal FBs were similar to those presented in a review by Marques et al. (1998)^1^, in order of preference the alligator forceps, Itard’s catheter and the Hartmann forceps. No patient required endoscopy or indirect laryngoscopy to remove oropharyngeal FBs. General anesthesia was required in only one case to remove an organic FB (bean) from the right ear, next to the tympanic membrane, in a 9-year-old child. The need for general anesthesia to remove FBs varies in literature, with percentages varying from 8.6% to 30%^5^.

Complications of ear FBs were seen in 8 patients (14.03%), lower than reported in literature.1 In this group 7 patients presented acute external otitis. This situation generated the debate of whether external otitis was a result of the FB duration in the external acoustic canal or if infection in the first place had led the patient to place a FB in the ear to relieve symptoms. External otitis was the most frequent complication in the review by Bressler et al. (1993), with a 7.1% incidence^4^. Laceration of the external auditory canal was seen in only 1 case and there was no perforation of the tympanic membrane. Five nose FB cases (38.46%) developed complications: 3 cases of rhinosinusitis, one case of epistaxis and 1 rhinolith. The complication rate for nose FBs was higher than other reports in literature^1^. No complications were seen in oropharyngeal FB cases.

Of the 13 cases with complications, 7 (53.85%) had undergone prior FB removal attempts in the hands of unprepared professionals or lay persons. In a review by Schulze et al. (2002) ear FB complications were significantly higher in patients who had undergone unsuccessful FB removal attempts^2^. This is similar to the findings in Bressler’s et al. review (1993), in which laceration of the external acoustic canal was seen in 61.5% of cases which had undergone unsuccessful FB removal attempts. This may be compared with the 5.1% complication rate in patients with no previous FB removal attempt^4^, underlining the importance of a ENT specialist to deal with these cases.

## CONCLUSION

Oropharyngeal foreign bodies were more frequent in patients over 20 years of age. The main symptom was odinofagia, which motivated an earlier search for medical services. Ear foreign bodies were more frequent in adults, with cotton as the most common material. Nose foreign bodies were more frequent in the 0-3 year age group and the main symptom was unilateral rhinorrhea.

The majority of cases in which unsuccessful foreign body removal attempts by untrained professionals or lay persons had been made developed complications, emphasizing the need for an ENT specialist with adequate instruments to manage such cases.
